# The Neuromuscular Junction and Wide Heterogeneity of Congenital Myasthenic Syndromes

**DOI:** 10.3390/ijms19061677

**Published:** 2018-06-05

**Authors:** Pedro M. Rodríguez Cruz, Jacqueline Palace, David Beeson

**Affiliations:** 1 Nuffield Department of Clinical Neurosciences, University of Oxford, Oxford OX3 9DU, UK; Jacqueline.palace@ndcn.ox.ac.uk (J.P.); David.beeson@ndcn.ox.ac.uk (D.B.); 2 Neurosciences Group, Weatherall Institute of Molecular Medicine, University of Oxford, The John Radcliffe Hospital, Oxford OX3 9DS, UK

**Keywords:** congenital myasthenic syndromes, neuromuscular junction, neuromuscular transmission, presynaptic CMS, COL13A1, SNARE complex, *N*-glycosylation pathway, GMPPB, β2-adrenergic agonists

## Abstract

Congenital myasthenic syndromes (CMS) are genetic disorders characterised by impaired neuromuscular transmission. This review provides an overview on CMS and highlights recent advances in the field, including novel CMS causative genes and improved therapeutic strategies. CMS due to mutations in *SLC5A7* and *SLC18A3*, impairing the synthesis and recycling of acetylcholine, have recently been described. In addition, a novel group of CMS due to mutations in *SNAP25B*, *SYT2*, *VAMP1*, and *UNC13A1* encoding molecules implicated in synaptic vesicles exocytosis has been characterised. The increasing number of presynaptic CMS exhibiting CNS manifestations along with neuromuscular weakness demonstrate that the myasthenia can be only a small part of a much more extensive disease phenotype. Moreover, the spectrum of glycosylation abnormalities has been increased with the report that *GMPPB* mutations can cause CMS, thus bridging myasthenic disorders with dystroglycanopathies. Finally, the discovery of *COL13A1* mutations and laminin α5 deficiency has helped to draw attention to the role of extracellular matrix proteins for the formation and maintenance of muscle endplates. The benefit of β2-adrenergic agonists alone or combined with pyridostigmine or 3,4-Dyaminopiridine is increasingly being reported for different subtypes of CMS including AChR-deficiency and glycosylation abnormalities, thus expanding the therapeutic repertoire available.

## 1. Introduction

**Figure 1:**
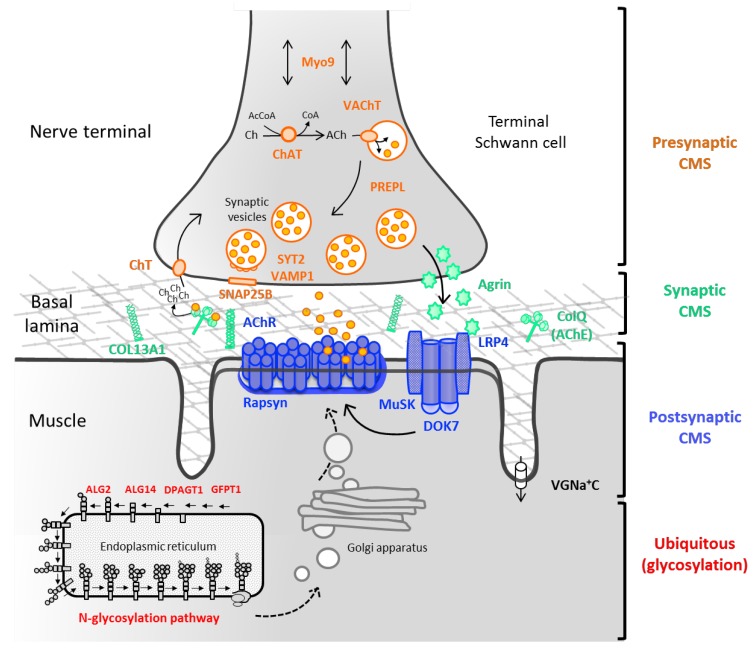
Schematic of the neuromuscular junction (NMJ) and the main molecules involved in congenital myasthenic syndromes. CMS result from presynaptic (ChAT, ChT, MUNC13-1, MYO9, PREPL, SYT2, VAChT, and VAMP1), synaptic basal lamina (COLQ and COL13A1), and postsynaptic defects (AChR subunits: α, β, δ and ε, AGRN, DOK7, MUSK, LRP4, and rapsyn). An increasing number of presynaptic CMS is being reported due to abnormalities in the synthesis, recycling or release of acetylcholine (normal arrows). The Agrin-LRP4-MuSK signaling pathway (bold arrows) is crucial for the clustering of the AChRs at the postsynaptic muscle membrane. Novel genes encoding for ubiquitous molecules (GFPT1, DPAGT1, ALG2, ALG14, and GMPPB) are represented in the endoplasmic reticulum (ER) in a simplified view of the *N*-glycosylation pathway. Post-translational modifications of the saccharide structure of the AChR and other NMJ proteins take place at the ER and Golgi apparatus (dashed arrows), before reaching the muscle cell surface as mature proteins. ACh, acetylcholine; AChE, acetylcholinesterase; AcCoA, acetyl coenzyme A; Ch, choline; VGNa + C, voltage-gated sodium channel.

Congenital myasthenic syndromes (CMS) are genetic disorders characterised by impaired neuromuscular transmission [1]. CMS is a rare condition with an estimated prevalence of genetically confirmed cases of approximately 9.2 cases per million children under 18 years of age in the UK [2]. All subtypes of CMS share the clinical feature of fatigable muscle weakness, but age at onset, presenting symptoms, distribution of weakness, and response to treatment vary, depending on the molecular mechanism that results from the underlying genetic defect. Most common classification of CMS relies on the location of the mutated protein (Figure 1).

The clinical diagnosis of CMS is based on the presence of fatigable muscle weakness (usually from an early age) in conjunction with abnormal findings on neurophysiological studies, in particular decremental response greater than 10% on repetitive nerve stimulation (RNS) or abnormal jitter and/or blocking on single-fibre electromyography (SFEMG), and positive response to pharmacological treatment. It is important to understand that although most patients have symptoms from birth or early childhood, some do present later in life during teenage years or adulthood. The classic myasthenic phenotype exhibits weakness of the ocular and facial muscles, but certain subtypes can present with apnoeic episodes or isolated limb-girdle weakness, thus increasing the diagnostic challenge.

Testing for antibodies against the acetylcholine receptor (AChR) and the muscle-specific kinase (MuSK) is useful to rule out myasthenia gravis (MG). However, negative results do not always exclude the presence of antibodies to the neuromuscular junction (NMJ) since novel targets have been recently identified [3,4] and antibody testing is not always readily available. An additional challenge are patients whose AChR antibody titre is negative on the classic radioimmunoprecipitation assay (RIA) but are positive for antibodies to clustered AChRs using a cell-based assay (CBA) [5]. Further complexity is encountered where congenital myopathies associate with secondary neuromuscular transmission abnormalities [6].

**Figure 2:**
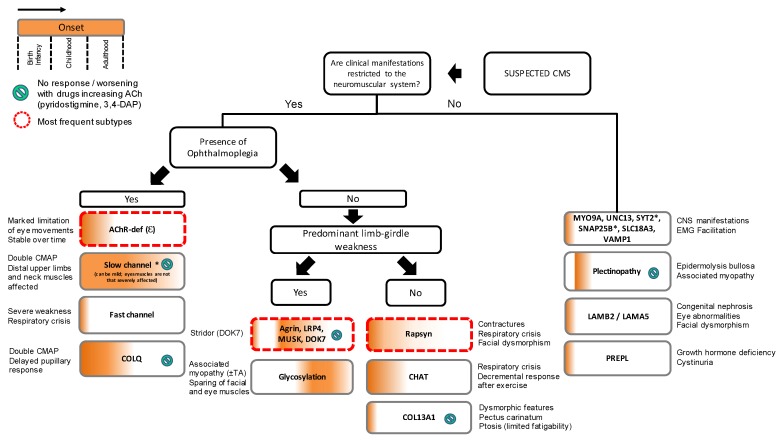
Proposed algorithm for targeted genetic screening of suspected CMS cases. Clinical evaluation should start by exploring age at onset and presence of manifestations beyond the neuromuscular boundaries. Ophthalmoplegia and limb-girdle weakness are clinically useful to guide genetic screening. Key diagnostic features are provided outside the boxes. Most frequent subtypes of CMS include AChR-deficiency, DOK7 CMS, and rapsyn CMS which stand for approximately 70% of all cases in the UK. (*) Slow channel syndrome, SYT2 CMS, and SNAP25B CMS are dominantly inherited. CNS, central nervous system; TA, tubular aggregates.

The genetic diagnosis of CMS is complex due to the existence of more than 30 CMS causative genes identified to date (Table 1). This number is likely to increase in the future due to the wider availability of next-generation sequencing (NGS). Features of the phenotype that help to tailor genetic screening include age at onset, distribution of weakness, electromyographic findings, and response to treatment (Figure 2). Recent insight has shown that patients with presynaptic CMS and with abnormalities within the glycosylation pathway may manifest with symptoms beyond the neuromuscular boundaries. Providing the appropriate symptomatic treatment requires the genetic subtype to be identified because treatments useful in some CMS subtypes can make other patients weaker. Furthermore, identifying a genetic cause for myasthenic weakness can help to avoid unnecessary immunosuppression and thymectomy.

## 2. Presynaptic Syndromes

**Figure 3:**
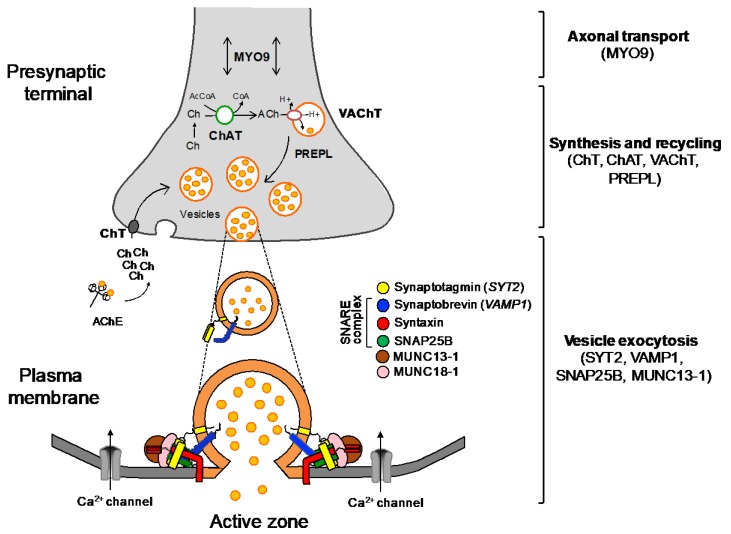
Schematic representation of the nerve terminal and the main molecules involved in presynaptic CMS. In the synaptic cleft, acetylcholinesterase (AChE) breaks down acetylcholine (ACh) into acetate and choline (Ch), which is uptaken by the sodium-dependent high-affinity choline transporter 1 (ChT) to the presynaptic terminal. The enzyme choline acetyltransferase (ChAT) catalyses the synthesis of ACh from acetyl coenzyme A (AcCoA) and choline, and the vesicular acetylcholine transporter (VAChT) loads ACh into synaptic vesicles. *PREPL* encodes a protein that is meant to act as an effector of the clathrin-associated adaptor protein 1 in the trafficking of VAChT [7]. The synaptic vesicles accumulate adjacent to the nerve terminal ready for exocytosis. Upon the arrival of an action potential, voltage-dependent Ca^2+^ channels open and the influx of Ca^2+^ cause the fusion of vesicles to the plasma membrane through the soluble *N*-ethylmaleimide-sensitive factor attachment protein receptor (SNARE) complex (synaptobrevin, syntaxin, and SNAP25B) and the Ca^2+^ sensor, synaptotagmin. Additionally, MUNC 13-1 and MUNC 18-1 (syntaxin-binding protein 1) take part in the assembly and disassembly of the complex through mechanisms still not fully understood [8]. Myosin-IX A is believed to be involved in axonal transport (two directions arrow). Adapted from [9].

The number of CMS subtypes caused by mutations in genes encoding presynaptic proteins has expanded in recent years thanks to the use of NGS (Figure 3). These can be subdivided depending on the pathogenic mechanism into disorders affecting axonal transport, the synthesis and recycling of ACh, and the exocytosis of synaptic vesicles. A considerable proportion of patients in this group present with early onset severe disease, episodic apnoeas, and central deficits derived from the expression of the encoded proteins within the central nervous system. This represents a novel aspect in CMS, where previously clinical manifestations were considered to be largely restricted to the NMJ.

### 2.1. Axonal Transport

#### MYO9A

*MYO9A* encodes myosin-IXA, which belongs to the superfamily of unconventional myosins [10]. These proteins are expressed in peripheral neurons and might play a role in axonal transport [11]. A recent study has reported three patients from two kinships with missense heteroallelic mutations in *MYO9A* [12]. All patients had severe neonatal onset with ptosis, hypotonia, and respiratory and bulbar involvement. Additional features included developmental delay, nystagmus and oculomotor apraxia. Treatment with pyridostigmine and 3,4-diaminopyridine was beneficial. Knockdown of MYO9A in zebrafish produced defects in neuronal branching and axon guidance suggesting a role in the integrity of the presynaptic terminal.

### 2.2. Synthesis and Recycling of Acetylcholine

#### 2.2.1. ChAT

Until recent years, mutations in *CHAT* were the only cause of presynaptic CMS. The enzyme choline acetyltransferase (ChAT) is responsible for the synthesis of acetylcholine from acetyl coenzyme A and choline in cholinergic neurons. The pathogenic mechanisms of *CHAT* mutations include low expression of ChAT, abnormal catalytic efficiency and compromise in thermal stability [13]. There are no apparent abnormalities in the NMJ structure [14]. The classic phenotype is neonatal onset CMS with life-threatening apnoeic crisis [15]. Some patients develop cerebral atrophy, most likely related to hypoxic episodes, although the effect of ChAT deficiency in the CNS cannot be ruled out. More recently, patients with onset of apnoeic episodes during infancy or early childhood and a milder course between crisis have been reported [16]. Treatment with pyridostigmine might help to prevent apnoeic episodes.

#### 2.2.2. PREPL Deficiency

The prolyl-endopeptidase-like gene (*PREPL*) encodes a protein that belongs to the prolyl-oligopeptidase subfamily of serine peptidases [17]. PREPL is ubiquitously expressed, although highest levels are reported in the brain, kidney, and muscle [18]. PREPL acts as an effector of the clathrin-associated adaptor protein 1 in the trafficking of the vesicular ACh transporter [19]. Hypotonia-cystinuria syndrome (HCS) associated with combined mutations in *PREPL* and *SLC3A1* (a contiguous gene to *PREPL* on chromosome 2p21) comprises type A cystinuria, growth hormone deficiency, and fatigable muscle weakness [7]. To date, a single CMS patient due to isolated PREPL deficiency has been reported [20]. The subject had severe hypotonia and feeding difficulties at birth with positive response to AChE inhibitors. The endplate study revealed normal endplate geometry, AChR density and kinetics, but reduced postsynaptic response [20].

#### 2.2.3. SLC5A7

*SLC5A7* encodes the presynaptic sodium-dependent high-affinity choline transporter 1 (ChT), which uptakes choline to the presynaptic terminal after the breakdown of ACh by AChE in the synaptic cleft [21]. There is a single report to date of *SLC5A7* loss-of-function mutations in seven individuals from six unrelated families [22]. Four subjects had a neonatal onset CMS with episodic apnoeas and positive response to AChE inhibitors while two had a more severe disease with arthrogryposis, malformations, and early death. Three patients suffered from cognitive delay. Ultrastructural analysis showed the presence of small nerve terminals and empty synaptic gutters.

#### 2.2.4. SLC18A3

*SLC18A3* encodes the vesicular acetylcholine transporter (VAChT), which loads ACh into synaptic vesicles in neurons [23]. Mutations in *SLC18A3* were first reported in two patients with episodic apnoeas, bilateral ptosis, and ophthalmoplegia. [24]. Additional features included learning difficulties and left ventricular dysfunction. The individual compound heterozygous for p.Gly186Ala and a genomic deletion in *SLC18A3* was able to walk independently at age 14 years and had positive response to pyridostigmine. The individual homozygous for p.Asp298His lost independent ambulation at five years of age. A second report described two siblings carrying a homozygous p.Gly360Arg substitution characterised by extreme hypotonia, breathing difficulties, microcephaly, and developmental delay [25]. One sibling died from respiratory failure five days after birth, and the other needed constant mechanical ventilation.

### 2.3. Synaptic Vesicles Exocytosis

This is a novel group of CMS caused by mutations in genes encoding proteins involved in synaptic vesicles exocytosis. Most comprise the soluble *N*-ethylmaleimide-sensitive factor attachment protein receptor (SNARE) complex and related proteins [26], which are involved in the docking and Ca^2+^ triggered fusion of synaptic vesicles with the presynaptic membrane at both central and neuromuscular synapses. Therefore, is not surprising that patients exhibit evident central manifestations and it is debatable whether myasthenia should really be their defining characteristic since it is only a small part of a severe wider phenotypic spectrum. Clinical neurophysiology can show post-exercise amplitude facilitation as seen in LEMS [27].

#### 2.3.1. SNAP25

*SNAP25* codifies the synaptosomal-associated protein 25, a core element of the SNARE-complex [28,29]. A single case of SNAP25 deficiency causing CMS has been reported to date [30]. The patient harboured the p.Ile67Asn de novo dominant mutation that was shown to inhibit synaptic vesicle exocytosis in vitro. The patient had multiple contractures and breathing difficulties at birth, achieving limited walking from seven years of age. Additional features included severe developmental delay, cortical hyperexcitability, and ataxic gait. Treatment with 3,4-DAP was beneficial.

#### 2.3.2. SYT2

*SYT2* encodes the synaptic vesicle membrane protein Synaptotagmin 2, which serves as a Ca^2+^ sensor for the exocytosis of synaptic vesicles [31]. Dominant mutations in *SYT2* (p.Asp307Ala and p.Pro308Leu) were first reported in two kinships featuring a non-progressive motor neuropathy and a presynaptic syndrome resembling LEMS [32]. The individuals suffered from foot deformities from childhood and a variable degree of proximal and distal weakness that improved with rest. Treatment with 3,4-DAP produced clinical and neurophysiological improvement [33].

#### 2.3.3. VAMP1

*VAMP1* encodes the vesicle associated membrane protein 1 (synaptobrevin 1), which is part of the SNARE complex, and has an essential role in Ca^2+^-triggered ACh release at the NMJ [34]. synaptobrevin 1 is also expressed in the brain, but in a less abundant manner than the highly homologous isoform synaptobrevin 2 [35]. Only four patients with CMS due to *VAMP1* mutations have been reported to date in two different kinships [36]. All patients suffered from a severe condition with hypotonia, muscle weakness and feeding difficulties at birth. Symptoms improved on pyridostigmine.

#### 2.3.4. UNC13A1

*UNC13A1* encodes MUNC13-1 (mammalian uncoordinated-13) protein, which plays a role in the priming of synaptic vesicles into a fusion competent state [37]. The only patient with *UNC13A1* mutations reported to date was homozygous for p.Gln102*, which eliminates the syntaxin 1B binding site of Munc13-1 [38]. The patient had a very severe phenotype with profound hypotonia and permanent need of ventilator support, and died at age 50 months from respiratory failure. There was no apparent clinical response to pyridostigmine or 3,4-DAP. Additional features included the presence of an abnormally thin corpus callosum in brain magnetic resonance imaging.

## 3. Synaptic and Basal-Lamina Associated Syndromes

**Figure 4:**
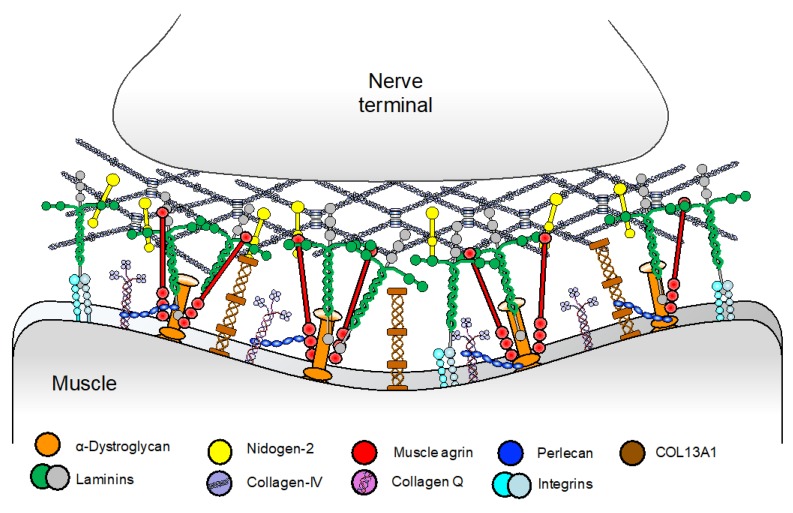
Schematic representation of the synaptic basal lamina and its main components. Laminins are heterotrimeric proteins of high molecular weight formed by the incorporation of α, β, and γ chains. Laminins self-assemble, but also interact with integrins and α-dystroglycan [41]. Collagen IV, which is the most abundant protein at the basal lamina, self-assembles into dimers and hexamers thanks to its globular domains [41]. Nidogen-2 are non-collagenous glycoproteins responsible for linking collagen IV and laminin networks [42,43]. Additional collagens include COL13A1 [44] and COLQ, a collagen like tail responsible for anchoring AChE to the synaptic cleft. Muscle agrin binds to the basal lamina via laminin [45] and α-dystroglycan [46], and this is important for maintenance of the NMJ [47]. This differs from the role of neuronal agrin as a key organiser of the postsynaptic apparatus via the AChR clustering pathway [48]. Perlecan, another synaptic heparan sulphate proteoglycan is linked to both ColQ [49] and α-dystroglycan [50].

The basal lamina of the NMJ is a structured form of extracellular matrix located at the synaptic cleft that is essential for the alignment, organization, and maintenance of pre- and postsynaptic structures [39]. The main components of the basal lamina are laminins, collagens, heparan sulfate proteoglycans (muscle agrin and perlecan), and nidogens [40] (Figure 4). Until recently, mutations in *COLQ* were the only CMS subtype in this category. Although other basal-lamina associated CMS have been recently reported, including laminin β2 and laminin α5 deficiencies and COL13A1 CMS, these are rare with only a few cases reported.

### 3.1. COLQ

*COLQ* encodes the collagen-like tail subunit of asymmetric acetylcholinesterase (AChE), which anchors AChE to the basal lamina. Mutations in *COLQ* cause endplate AChE deficiency [51]. It is likely that neuromuscular transmission in COLQ CMS is impaired through different mechanisms. First, the prolonged time of ACh at the synaptic cleft produce desensitization of AChRs and secondary endplate myopathy with loss of AChRs due to sarcoplasmic Ca^2+^ overload [51]. Second, if *COLQ* mutations alter the interaction of ColQ with MuSK or perlecan, this could impact postsynaptic differentiation [52,53]. *COLQ* mutations have been reported in all domains of the protein with no phenotype–genotype correlation [54]. The classic phenotype is that of neonatal or early onset severe disease with ptosis, ophthalmoparesis, generalised weakness, respiratory difficulties, and progressive course [51]. Additional phenotypes include early onset disease with a mild course, and cases reminiscent of DOK7 CMS [54]. Repetitive CMAP from a single nerve stimulus can be seen due to prolonged endplate currents outlasting the refractory period of the muscle fibre [55]. Pyridostigmine and 3,4-DAP are contraindicated but some patients do respond to β2-adrenergic agonists [56].

### 3.2. COL13A1

*COL13A1* encodes the α chain of a non-fibrillar collagen, which has shown in vitro binding with other extracellular matrix proteins such as integrin α1β1 [57], nidogen, and perlecan [58]. Studies in animal models lacking COL13A1 identified abnormal maturation of the neuromuscular junction [44]. *COL13A1* mutations have been reported in two unrelated families with CMS [59]. Onset of symptoms was at birth with breathing and feeding difficulties. Bilateral ptosis was present and eye movements were normal. The analysis of the muscle biopsy in the individual carrying the c.1171delG loss-of function mutation showed loss of COL13A1 expression at the muscle endplate. Functional analysis demonstrated a deleterious effect on AChR clustering in vitro. Treatment with 3,4-DAP and salbutamol was beneficial. The second family carried the homozygous splice site mutation c.523-1delG (p.Leu392fs*71), which is predicted to lead to premature termination (p.Gly175Vfs*20). Overall, these findings draw attention to the role of extracellular matrix proteins for the formation and maintenance of the NMJ. The beneficial effect of salbutamol would be consistent with *COL13A1* mutations affecting AChR clustering and maturation of postsynaptic structures. The positive effect of 3,4-DAP is supported by the presynaptic abnormalities seen in the *Col13a1*^−/−^ mouse model such as abnormal clustering of synaptic vesicles in the motor nerve terminal.

### 3.3. Laminin β2 and Laminin α5 Deficiencies

A single CMS case of laminin β2 deficiency due to heteroallelic frameshift mutations in *LAMB2* has been reported to date [60]. The neuromuscular phenotype was characterised by neonatal onset with breathing difficulties, ptosis, ophthalmoplegia, and profound proximal weakness. Cholinesterase inhibitors were deleterious in this single case. Additional features included congenital nephrosis and distinct eye abnormalities with microcoria (Pierson syndrome) [61] in keeping with Laminin β2 being an important component of the glomerular basement membrane and intraocular muscles.

Homozygous missense mutations in *LAMA5*, encoding laminin α5, have been reported in a neonatal onset CMS combining myopia, facial tics, and abnormal neuromuscular transmission [62]. The endplates study found normal postsynaptic membrane but small nerve terminals. Neurophysiology showed abnormalities in keeping with a LEMS-like presynaptic defect. There was a positive response to treatment with pyridostigmine and 3,4-diaminopyridine.

## 4. Postsynapatic Syndromes

### 4.1. Primary AChR Deficiency

**Figure 5:**
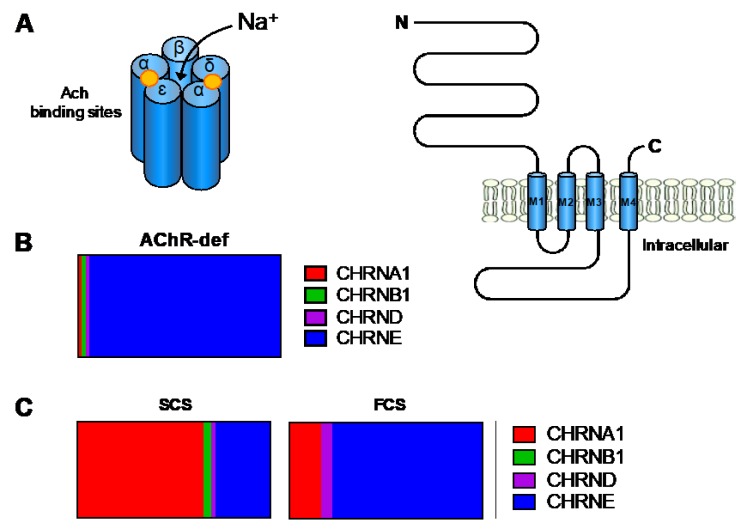
The adult AChR and the genetics of AChR-deficiency and kinetics abnormalities of the AChR. (**A**) The AChR is made up of five subunits organised around a central pore. Each subunit is composed of an extracellular domain, four transmembrane domains (M1–M4), and a large cytoplasmic loop that links M3 and M4; (**B**,**C**) Relative proportion of genetic defects in patients with AChR deficiency and kinetic abnormalities of the AChR within the Oxford CMS cohort. AChR deficiency is mainly caused by mutations in *CHRNE* encoding the *ε*-subunit of the AChR. SCS is often caused by mutations in *CHRNA1* encoding the AChR α-subunit, while FCS is most commonly due to mutations in *CHRNE*.

The adult nicotinic AChR is a pentameric complex composed of four different transmembrane subunits (2 α, β, δ, and ε-subunits) encoded by *CHRNA1*, *CHRNB1*, *CHRND*, and *CHRNE*, respectively (Figure 5A). Primary AChR deficiency is characterised by reduced AChR numbers (10 to 30% of normal values) and integrity of the postsynaptic folding [63]. This CMS subtype is mainly caused by *CHRNE* mutations (Figure 5B) and accounts for approximately 30% of total CMS in the UK. These are found along the entire gene including the promoter region [64] and result in protein truncation, loss of essential residues (glycosylation sites, Cys-loop) for AChR assembly or function [65], or severely reduced levels of subunit mRNA expression. It is believed that incorporation of the foetal AChR γ-subunit into the AChR pentamer enables subjects with null ε-subunits alleles to survive [66]. The phenotypic spectrum of patients with primary AChR-deficiency is wide from mild to severe disease [64]. Most patients have a marked limitation of ocular movements, which is useful to guide genetic screening. Treatment with cholinesterase inhibitors and 3,4-DAP is beneficial, although the long-term response in severe cases is often incomplete [67]. Some patients may have dramatic responses to the addition of β2-adrenergic agonists [68].

### 4.2. Kinetic Abnormalities of the AChR (with or without AChR Deficiency)

Mutations in any of the four AChR adult subunits can also alter ion channel function leading to prolonged (slow channel syndrome, SCS) or abbreviated openings (fast channel syndrome, FCS). However, mutations in *CHRNA1* and *CHRNE* are the most frequent (Figure 5C). This group represents approximately 15% of total CMS cases in the UK.

SCS was the only CMS with autosomal dominant inheritance until the recent descriptions of CMS due to *SNAP25* and *SYT2* mutations. The underlying pathogenic mechanism is prolonged AChR opening, which causes desensitisation of AChRs, depolarisation block, and secondary endplate myopathy due to cationic overload [69] and focal activation of caspases [70]. The age of symptom onset in SCS is very variable, from birth to the fifth decade, and patients are in general not as severely affected as other subtypes. Ptosis and ophthalmoparesis can be present but to a lesser degree than in primary AChR deficiency. There is often selective impairment of cervical and distal upper limb muscles. Treatment is with AChR open channel blockers such as fluoxetine or quinidine [71]. Drugs increasing ACh levels can be deleterious.

FCS result in brief channel openings with secondary reduced postsynaptic depolarisation and failure to trigger muscle action potentials [72]. Many FCS are characterised by severe weakness from birth, ptosis, ophthalmoplegia, and life-threatening respiratory crises that may be fatal [73]. FCS is rare because, in order to define the clinical phenotype, the FCS mutation needs to be homozygous or in compound heterozygosis with a null or low expressor mutation. The εP121L variant, which is critical for binding of acetylcholine [74], is the most common FCS mutation in the UK [73]. Pyridostigmine and 3,4-DAP are beneficial, although the effect may decrease with time.

### 4.3. Defects within the AChR-Clustering Pathway

**Figure 6:**
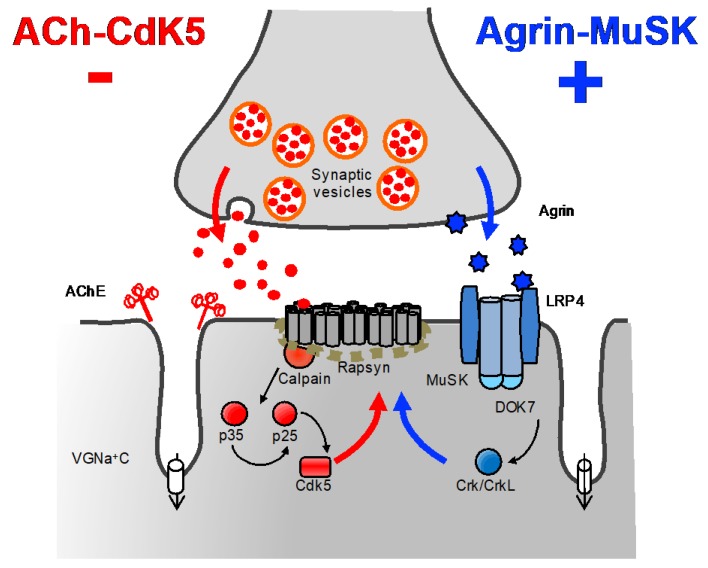
The agrin-induced AChR clustering and ACh-CdK5 dispersal pathways. The AChR clustering pathway is shown blue and the dispersal pathway in red. Main molecules with a role in synapse formation and maintenance are represented although additional, still unknown, positive and negative factors are very likely to be involved. CMS patients harboring mutations within the AChR clustering pathway (excluding *RAPSN*) present common clinical features such as relative sparing of eye muscles, predominant limb girdle weakness, worsening of symptoms with drugs increasing ACh levels, and improvement on long-term therapy with β2-adrenergic agonists. DOK7 CMS represents the most frequent subtype in this category with other syndromes being rather infrequent.

The AChR clustering signaling pathway is essential for the formation and maintenance of the NMJ [75] (Figure 6). Upon release of agrin by the nerve terminal, agrin binds to LRP4 at the postsynaptic membrane resulting in MuSK dimerisation and activation [76]. This leads to the recruitment of DOK7, a muscle-specific cytoplasmic adaptor of MuSK that further stimulates MuSK kinase activity propagating the signal downstream through mechanisms still not elucidated. It results in the phosphorylation of the AChR β-subunit, which promotes binding of the cytoplasmic anchoring protein rapsyn and final stabilisation of innervated AChR clusters [77]. Additional players (although their role is still poorly understood) include CrK/CrKL [78] and possibly Dishevelled [79], a scaffold protein involved in Wnt signaling pathways [80].

By contrast to the AChR clustering pathway, a negative signal disperses aneural AChR clusters not stabilised by agrin signaling (Figure 6). This pathway is thought to be driven by ACh through a cyclin-dependent kinase 5 (Cdk5) mechanism [81] linked to the interaction of rapsyn and the calcium-dependent protease calpain [82]. Calpain promotes the cleavage of p35 to p25, a potent activator of Cdk5 [83]. Rapsyn is believed to stabilise AChR clusters by suppressing calpain activity [82].

#### 4.3.1. AGRN

*AGRN* mutations account for a rare CMS subtype with only four reports published to date. The phenotypic spectrum is variable from very severe disease with respiratory failure [84] to a mild CMS with running difficulties [85], limb-girdle weakness [86], or dropped head presentation [87]. Overall, there is no clear phenotype–genotype correlation, including a recent report of five patients from three different kinships with a similar phenotype comprising CMS with distal muscle weakness and atrophy [88]. Of note, extraocular muscles were usually spared or modestly affected in keeping with other CMS impairing the AChR clustering pathway. Most patients were unresponsive to pyridostigmine and 3,4-DAP, but some had a clear benefit on β2-adrenergic agonists [88].

#### 4.3.2. LRP4

The nerve terminal releases agrin that binds to the low-density lipoprotein receptor-related protein 4 (LRP4) forming a ternary complex with MuSK that triggers MuSK phosphorylation [76,89]. CMS due to *LRP4* mutations is extremely rare with only three patients from two different kinships reported to date [90,91]. The clinical features of the few patients reported were variable with one case of early onset severe myasthenia and two milder cases with onset in childhood after normal or slightly delayed motor milestones. In a similar fashion to DOK7 CMS, patients improved on β2-adrenergic agonists while pyridostigmine worsened muscle weakness. All patients harboured missense mutations located at the third propeller domain of LRP4 that prevent MuSK activation. Mutations in other domains of LRP4 may cause Cenani–Lenz syndrome.

#### 4.3.3. MuSK

*MUSK* encodes the muscle specific kinase (MuSK), a key element of the agrin signalling pathway [92]. MuSK is composed of three IgG-like domains, a frizzled domain, and a kinase domain. *MUSK* mutations represent a very rare cause of CMS with only few cases reported [93,94,95,96]. However, given the role of MuSK and DOK7 in the same pathway, it is noteworthy that the clinical features and treatment response are similar in both conditions, with predominant limb girdle weakness, sparing of eye muscles, and worsening with cholinesterase inhibitors. A recent report suggests an increased likelihood of a severe, respiratory phenotype with null alleles, while late onset CMS may be associated with missense variants affecting the kinase domain (excluding the catalytic site) [96].

#### 4.3.4. DOK7

DOK7 is a muscle-specific cytoplasmic adaptor protein of MuSK, which is essential for postsynaptic specialization of the NMJ [97]. Mutations in *DOK7* underlie a NMJ synaptopathy [98], which accounts for approximately 15–20% of total CMS cases in the UK. The spectrum of *DOK7* mutations is wide although most patients have at least one mutation within the C-terminus, with c.1124_1127dupTGCC present in approximately 65% of the cases [99]. Although patients occasionally may present in adulthood, the classic phenotype is characterised by onset of symptoms in childhood after normal motor milestones, with progressive limb-girdle weakness and walking difficulties [98]. Patients have ptosis but eye movements are typically normal. The clinical spectrum varies from mild limb-girdle weakness to generalised and severe weakness. There is no clear genotype-phenotype correlation [100]. Patients can improve dramatically on β2-adrenergic agonists in the course of months [101].

### 4.4. Rapsyn Deficiency

*RAPSN* encodes the 43 kDa receptor-associated scaffold protein of the synapse [102], which is essential for stabilisation of AChR clusters at the muscle endplate [103,104]. Rapsyn is enriched at postsynaptic membranes and acts as a linker between the AChRs and the cytoskeleton via the dystrophin-associated glycoprotein complex [105]. The detailed organisation of the AChRs-rapsyn network is not fully understood since the crystallographic structure has not been solved [106].

Rapsyn CMS is characterised by deficiency of AChRs at the postsynaptic membrane and poor development of postjunctional folds [107]. *RAPSN* mutations have been reported across the entire length of the gene although most patients are either homozygous for p.N88K mutation or heteroallelic for p.N88K and a second mutation [108]. The major effect of p.N88K is to reduce the stability of AChR clusters [108]. Most patients with *RAPSN* mutations present at birth or early in life with generalised hypotonia, respiratory weakness, and feeding difficulties [109,110]. Mild arthrogryposis, facial dysmorphism, ptosis, and strabismus are usually present but ophthalmoplegia is rare. Life-threatening respiratory crises are frequent during infancy and early childhood. Additional phenotypes include lethal foetal akinesia [111] and a late onset milder disease [109]. Treatment with pyridostigmine and 3,4-DAP is beneficial. Most patients improve with age and have a good long-term prognosis [112].

## 5. CMS Due to Abnormal Glycosylation

**Figure 7:**
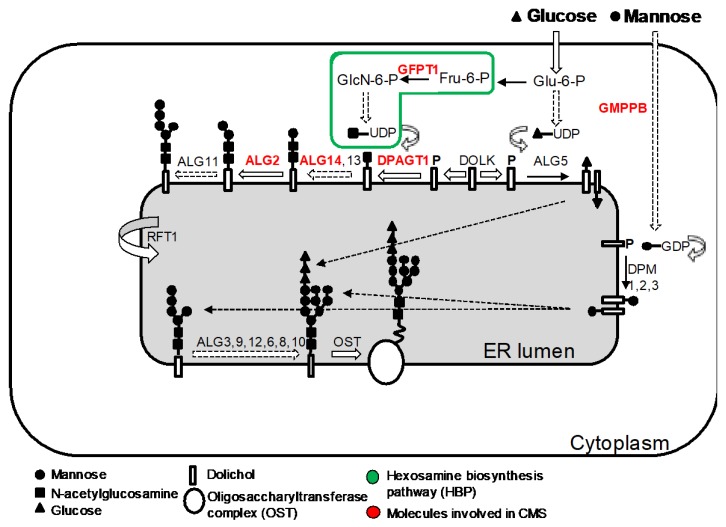
Simplified representation of the *N*-glycosylation pathway of proteins and the molecules involved in CMS. The *N*-linked glycosylation of proteins takes place in the ER. It starts with the assembly of the core glycan (*N*-acetylglucosamine, glucose and mannose) on the lipid dolichol. A series of cytosolic glycosyltransferases proceed to dolichol glycosylation on the cytoplasmic face of the ER: GFPT1 synthesizes UDP-GlcNAc (Uridine diphosphate *N*-acetylglucosamine); DPAGT1 and the ALG13/14 complex are involved in adding the first and second *N*-acetylglucosamine to dolichol. Additional sugar residues are added by ALG2 and other enzymes until the resulting product is flipped into the ER lumen by RFT1. Inside the ER lumen, sugar moieties are incorporated until the glycan is transferred to asparagine residues of nascent proteins by the multimeric oligosaccharyl transferase complex (OST) that subsequently will be modified inside the ER and Golgi. DOLK, dolichol kinase; DPM, dolichol-phosphate mannose synthase; Fru-6-P, fructose-6-phosphate; GlcN-6-P, glucosamine-6-phosphate; Glu-6-P, glucose-6-phosphate; GMPPB, GDP-mannose pyrophosphrylase B.

The *N*-linked glycosylation pathway is a ubiquitous process in eukaryote cells defined by the sequential attachment of sugar moieties to the lipid dolichol, which is then transferred to an asparagine residue in a protein (Figure 7). Mutations in components within this pathway produce a spectrum of severe multisystemic disorders known as congenital disorders of glycosylation [113,114,115]. In addition, NGS has aided the discovery of an unexpected relationship between glycosylation defects in the early stages of the *N*-glycosylation pathway and CMS [116,117,118]. The reasons why, in certain cases, defects in a ubiquitous process result in dysfunction largely restricted to the NMJ is unclear.

Glycosylation of AChR subunits is required for the correct assembly of AChR pentamers and for efficient export to the cell surface [119] and thus abnormal glycosylation results in reduced AChRs at the muscle endplates, which is most likely the primary mechanisms leading to impaired neuromuscular transmission [120].

These patients constitute a distinctive clinical group where muscle weakness is often confined to the limb girdles, and classic myasthenic manifestations—such as ptosis, ophthalmoplegia, or facial weakness—are not present. In relation to this, RNS and SFEMG of facial muscles are often normal causing diagnostic difficulties and thus neurophysiology should also be performed in proximal muscles. Concomitant myopathy is often present, which makes it a progressive condition over time. The myasthenic component can be treated with a combination of pyridostigmine, 3,4-DAP and salbutamol. Intellectual disability is also seen in some cases. The description of this novel group of disorders points that CMS should be part of the differential diagnosis of limb girdle muscle weakness.

### 5.1. GFPT1

*GFPT1* encodes glutamine-fructose-6-phosphate transaminase-1 (GFAT1). This enzyme catalyses the first step in the biosynthesis of UDP-*N*-acetylglucosamine, an essential substrate for *N*- and *O*-glycosylation of proteins [121]. GFPT1 knock-down in zebrafish embryos confirmed that GFPT1 is required for NMJ formation [117]. Mutations in *GFPT1* have been identified in more than 50 patients to date causing an autosomal recessive limb-girdle CMS [117,122,123,124]. The clinical presentation is usually with proximal muscle weakness and sparing of ocular and facial muscles. Clinical onset of symptoms is variable from early childhood to the second and third decades. If present, modestly elevated serum creatine kinase (CK) levels and additional myopathic changes on needle EMG suggest a concomitant myopathy [123]. Tubular aggregates (TA) are commonly found on muscle biopsy. The expression of α-dystroglycan is preserved. Treatment with AChE inhibitors and 3,4-DAP is beneficial [125].

### 5.2. DPAGT1

*DPAGT1* encodes the enzyme dolichyl-phosphate-*N*-acetylglucosamine-phophotranferase-1 that catalyses the first step in the dolichol oligosaccharide pathway for glycoprotein biosynthesis [126]. CMS due to *DPAGT1* mutations has been reported in 12 patients [127,128,129]. Presentation is during infancy or childhood with prominent limb-girdle weakness and minimal craniobulbar manifestations [130]. The spectrum of severity is variable but life-threatening crisis are rare [127]. A concomitant myopathy is present with or without tubular aggregates. Cognitive manifestations can vary from none to mild learning difficulties or major intellectual disability [128]. Endplate studies have shown pre- and postsynaptic abnormalities with reduction of postsynaptic folding, small nerve terminals, and reduced α-bungarotoxin labelling. Therapy with pyridostigmine and 3,4-DAP is usually beneficial. Of note, β2-adrenergic agonists had a clear benefit in two patients [128,130].

### 5.3. ALG2 and ALG14

*ALG14* is thought to form, together with ALG13 and DPAGT1, a functional multienzyme complex involved in the initial steps of *N*-linked protein glycosylation [131]. *ALG2* encodes alpha-1,3-mannosyltransferase that catalyses the second and third mannosylation steps for the elongation of the carbohydrate chain linked to dolichol [132]. Both ALG2 and ALG14 are concentrated at motor endplates and RNA silencing of ALG14 results in reduced cell-surface expression of AChRs in heterologous cells [118]. CMS due to mutations in *ALG2* and *ALG14* is very rare with only nine patients from three different kinships reported to date [118,133]. Clinical features are similar to those described in patients with GFPT1 and DPAGT1 CMS.

### 5.4. GMPPB

*GMPPB* encodes GDP-mannose pyrophosphorylase B that catalyses the conversion of mannose-1-phosphate and GTP to GDP-mannose. *GMPPB* contributes to both the *O*-mannosylation and *N*-glycosylation pathways. Mutations within the *O*-mannosylation pathway were originally identified in patients with dystroglycanopathies, a form of muscular dystrophy characterised by reduced α-dystroglycan glycosylation [134]. More recently, it was shown that mutations in *GMPPB* can also cause CMS and bridge myasthenic disorders with dystroglycanopathies [135]. Patients with GMPPB CMS have prominent limb-girdle weakness with minimal or absent craniobulbar manifestations [136]. Presentation is often delayed beyond infancy with proximal muscle weakness although patients often recall poor performance in sports during childhood. Muscle biopsy typically shows dystrophic features and reduced α-dystroglycan glycosylation. Myopathic changes can be present on muscle MRI and serum CK is significantly increased compared to other CMS subtypes. Patients are responsive to acetylcholinesterase inhibitors alone or combined with 3,4-diaminopyridine and/or salbutamol.

## 6. Treatment

**Figure 8:**
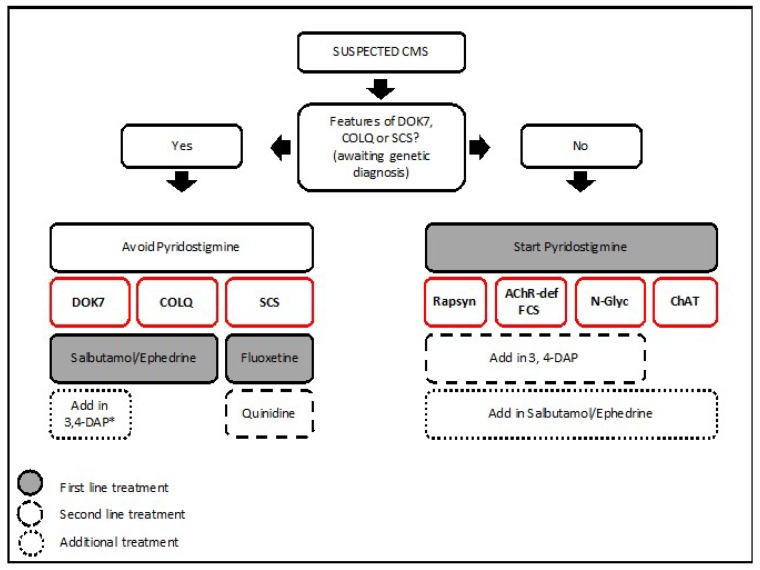
Treatment strategy at the Oxford CMS Service for most common CMS subtypes. Whenever features of DOK7 and other members of the AGRN–MUSK pathway, COLQ, or SCS are present, first-line treatment with pyridostigmine should be avoided until confirmation of genetic diagnosis. 3,4-DAP can be added in with caution in certain CMS subtypes (* use with caution) [137]. Combined therapy with β2-adrenergic agonists may be tried on AChR deficiency, CMS due to glycosylation defects, FCS, and rapsyn CMS. In cases of SCS, quinidine may be used as an alternative to fluoxetine. FCS, Fast channel syndrome; *N*-glyc, *N*-glycosylation pathway; SCS, slow channel syndrome.

The CMS treatment strategy used in the Oxford CMS Service is provided for the most common CMS subtypes (Figure 8). As shown in the figure, genetic diagnosis is crucial to provide adequate pharmacological treatment for every CMS subtype.

Classic treatments include acetylcholinesterase inhibitors (pyridostigmine–Mestinon®) to inhibit acetylcholinesterase from breaking down acetylcholine [138]; 3,4-DIaminopyridine (3,4-DAP) that works by blocking presynaptic potassium channels and thus increases the action potential duration and acetylcholine release [139]; fluoxetine and quinide work as open channel blockers to restore synaptic currents in slow channel syndrome [140].

A number of studies have reported the remarkable benefit of therapy with β2-adrenergic agonists such as salbutamol and ephedrine in DOK7 CMS [101,141,142]. The use of these drugs is increasingly being reported in other CMS subtypes such as AChR-deficiency [68], acetylcholinesterase deficiency [54,143], and CMS due to abnormal glycosylation [128,130]. The molecular mechanism for salbutamol and ephedrine at the NMJ is unknown. In patients with mutations in the AGR*N*-MUSK-DOK7 pathway, there is a slow but progressive and marked response starting within weeks and increasing in effect before stabilising at 6–24 months [101]. In patients with AChR-deficiency on pyridostigmine, the effect on muscle strength and fatiguabilty seems already significant within the first two weeks of starting treatment, although the improvement can continue until the first 6–12 months [68]. These observations suggest that the benefit of β2-adrenergic agonists do not derive from short-term effect on neuromuscular transmission, but from a long-term effect probably related to increased structural stability or remodeling of the muscle endplates. The development of more specific β2-adrenergic receptor agonists would be of interest to maximize the benefits of pharmacological treatment while avoiding generalized side effects derived from adrenergic stimulation.

## Figures and Tables

**Table 1:** CMS subtypes and associated genes

CMS Subtype	Gene
Proteins with defined NMJ function	
Presynaptic	
Choline *O*-Acetyltransferase	*CHAT*
Unconventional myosin 9	*MYO9A*
PREPL	*PREPL*
Vesicular ACh transporter (VAChT)	*SLC18A3*
High-affinity choline transporter 1 (ChT)	*SLC5A7*
Synaptosome Associated Protein 25	*SNAP25B*
Synaptotagmin 2	*SYT2*
MUNC13-1	*UNC13*-*1*
Synaptobrevin 1	*VAMP1*
Synaptic	
Collagen Type XIII α1 Chain	*COL13A1*
Endplate AChE deficiency	*COLQ*
Laminin α5 deficiency	*LAMA5*
Laminin β2 deficiency	*LAMB2*
Postsynaptic	
Agrin (neuronal)	*AGRN*
Primary AChR deficiency	*CHRNA*, *CHRNB*, *CHRND*, *CHRNE*
Slow channel syndrome	*CHRNA*, *CHRNB*, *CHRND*, *CHRNE*
Fast channel syndrome	*CHRNA*, *CHRNB*, *CHRND*, *CHRNE*
Low conductance syndrome	*CHRNE*
Escobar syndrome	*CHRNG*
DOK7	*DOK7*
LRP4	*LRP4*
MuSK	*MUSK*
Plectin deficiency	*PLEC1*
Rapsyn	*RAPSN*
Sodium channel myasthenia	*SCN4A*
Ubiquitously expressed proteins	
ALG2	*ALG2*
ALG14	*ALG14*
DPAGT1	*DPAGT1*
GFPT1	*GFPT1*
GMPPB	*GMPPB*
SLC25A1	*SCL25A1*

ALG2, α-1,3/1,6-mannosyltransferase; ALG14, UDP-*N*-acetylglucosaminyltransferase subunit; DOK7, docking protein 7; DPAGT1, dolichyl-phosphate *N*-acetylglucosaminephosphotransferase 1; GFPT1, glutamine-fructose-6-phosphate transaminase 1; GMPPB, GDP-mannose pyrophosphorylase B; LRP4, LDL receptor related protein 4; MuSK, Muscle specific kinase; PREPL, prolyl endopeptidase-like gene; SLC25A1, solute carrier family 25 member 1.
